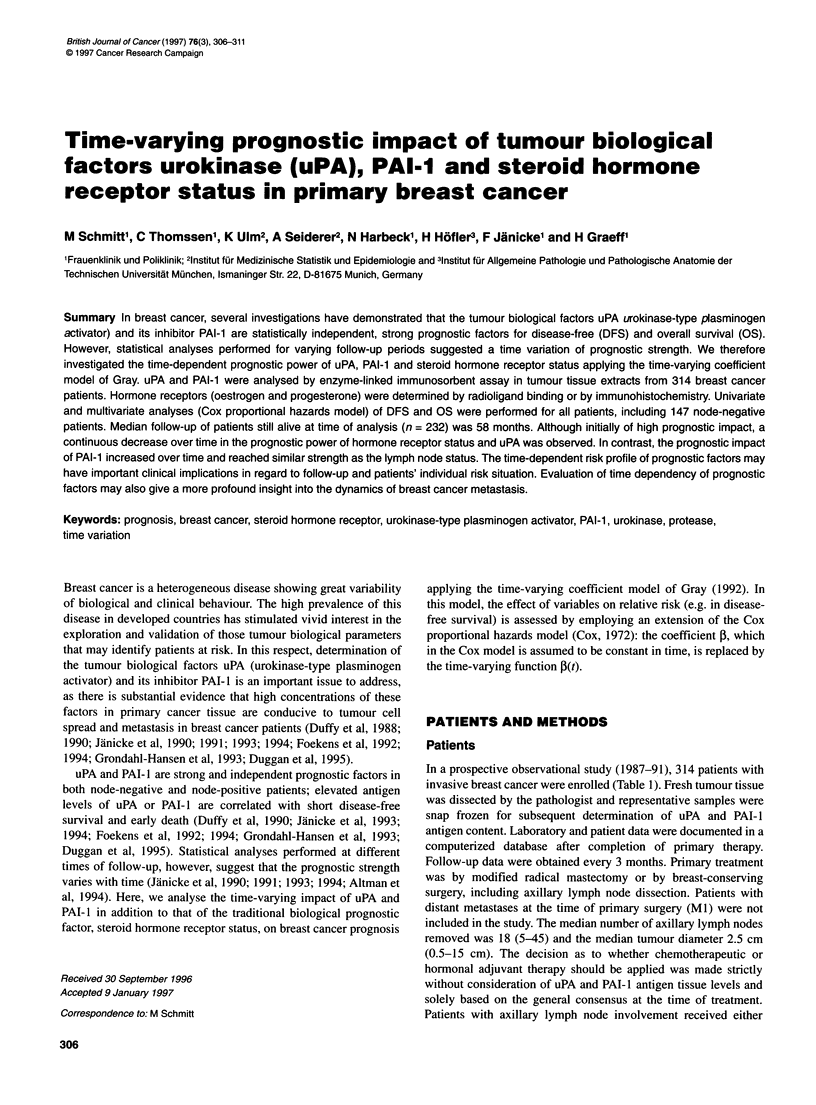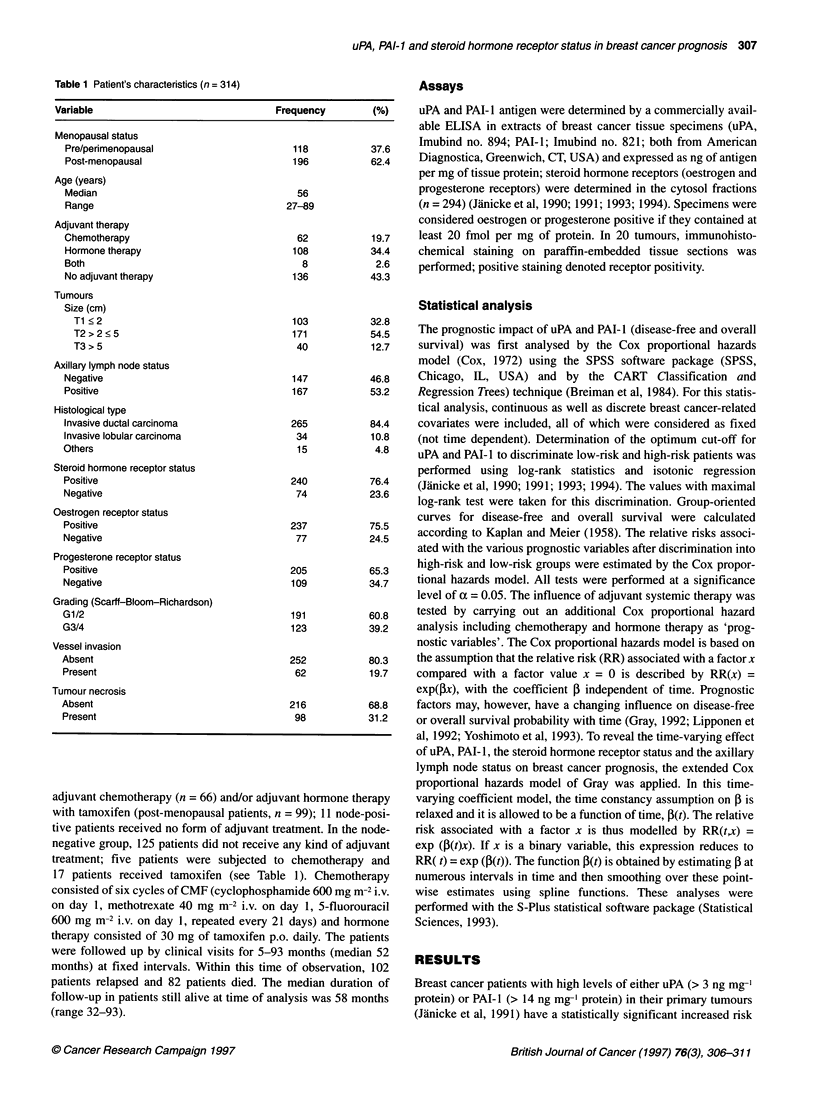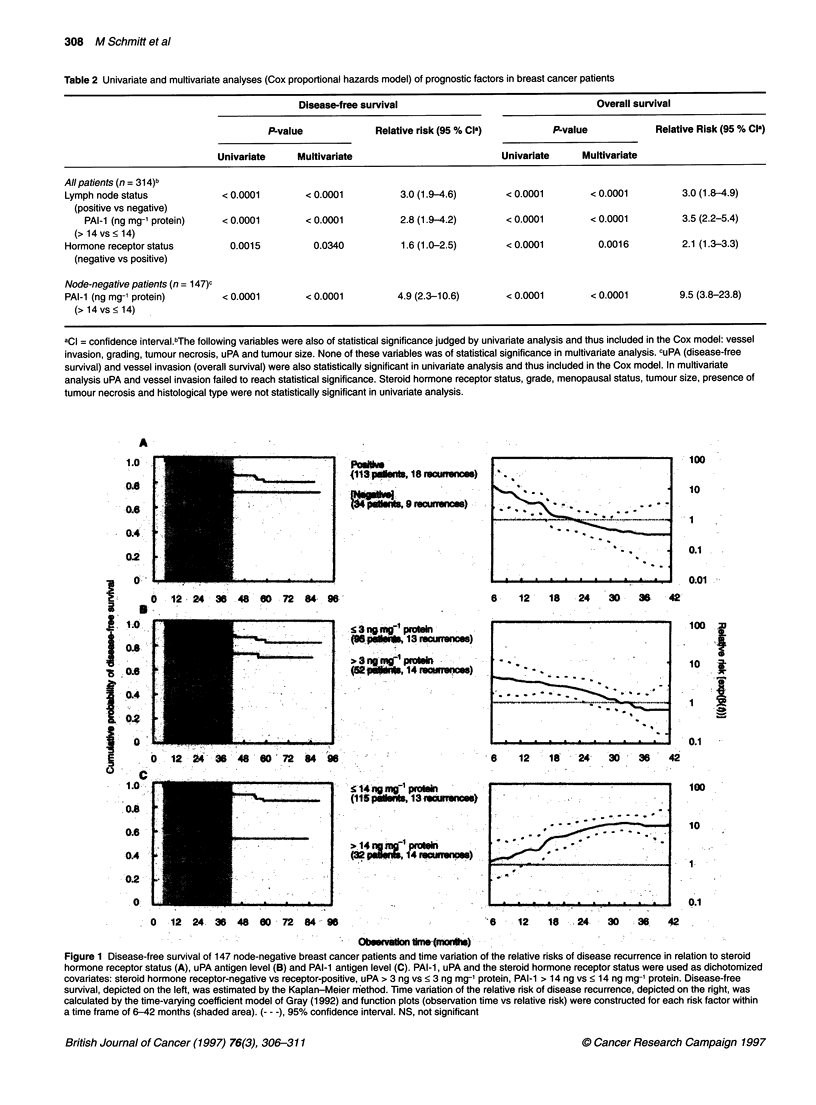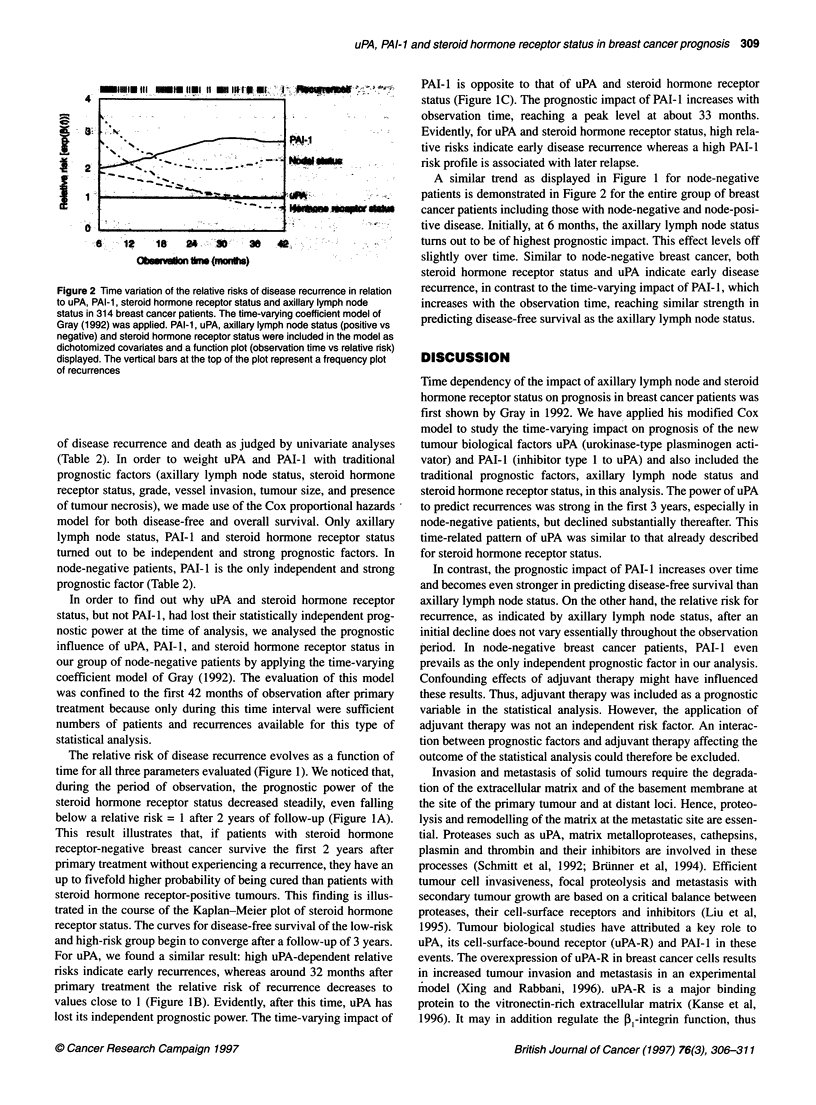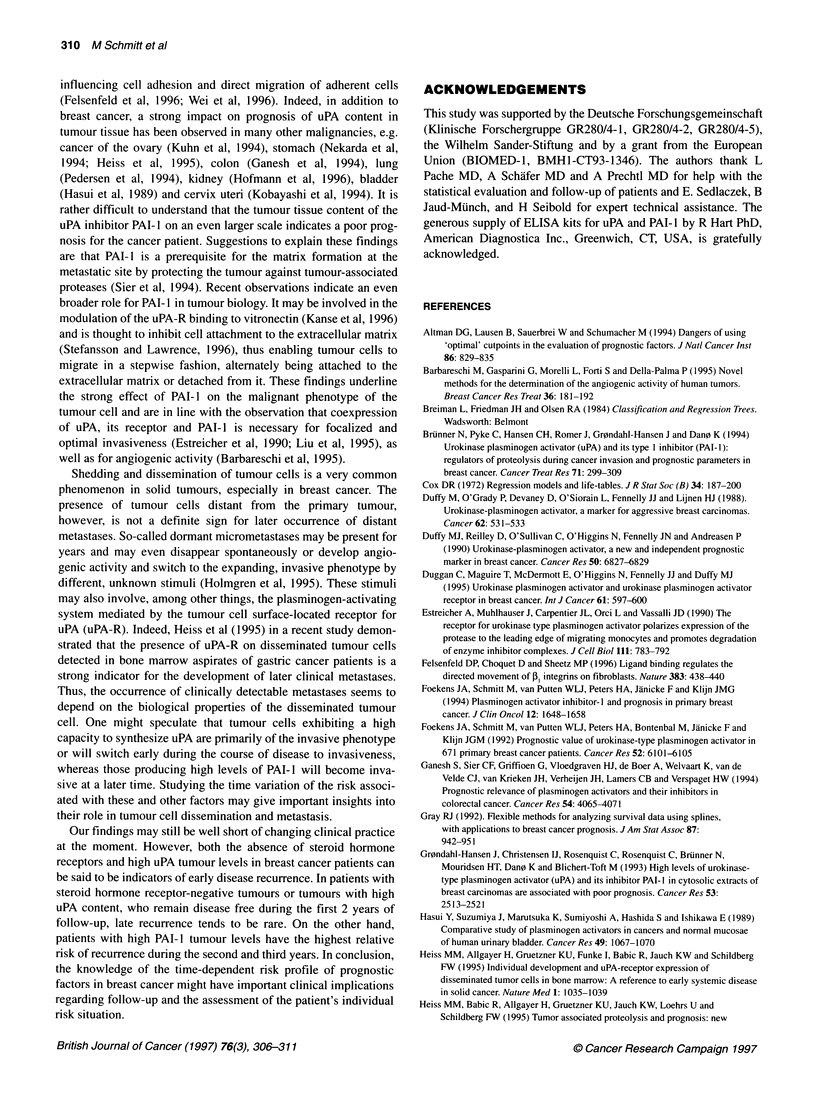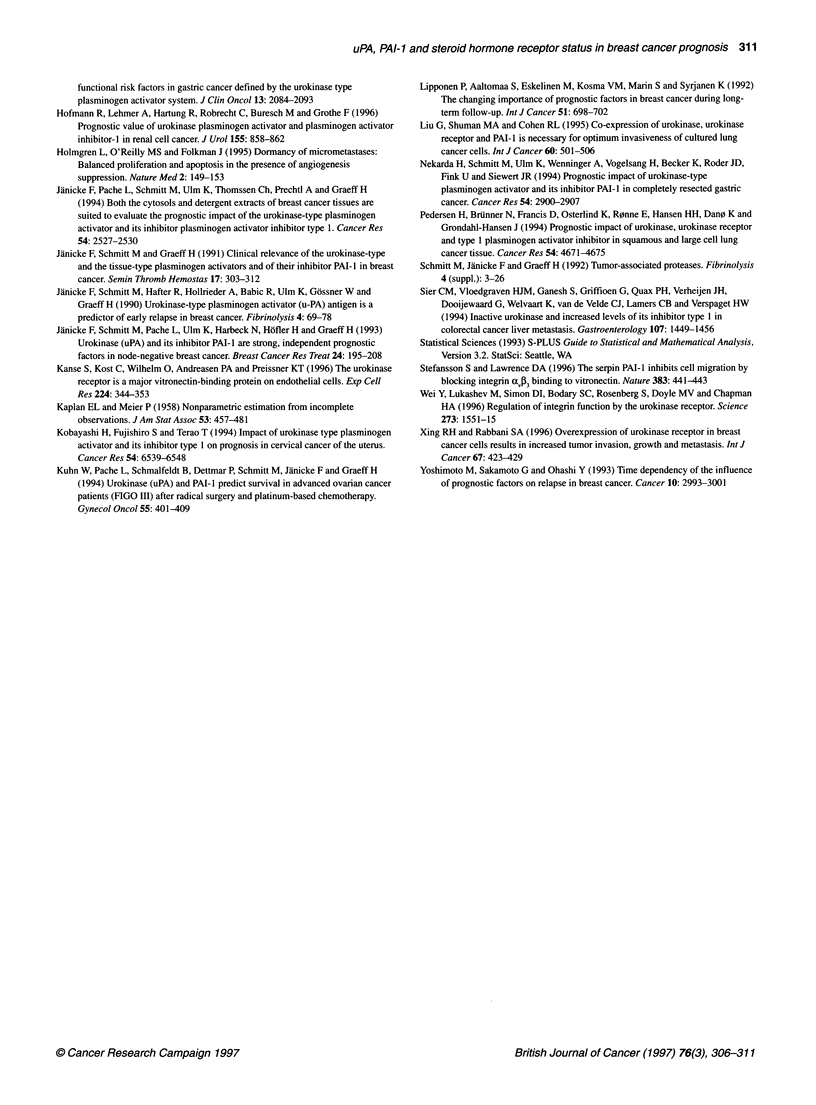# Time-varying prognostic impact of tumour biological factors urokinase (uPA), PAI-1 and steroid hormone receptor status in primary breast cancer.

**DOI:** 10.1038/bjc.1997.383

**Published:** 1997

**Authors:** M. Schmitt, C. Thomssen, K. Ulm, A. Seiderer, N. Harbeck, H. HÃ¶fler, F. JÃ¤nicke, H. Graeff

**Affiliations:** Frauenklinik und Poliklinik, Munich, Germany.

## Abstract

In breast cancer, several investigations have demonstrated that the tumour biological factors uPA urokinase-type plasminogen activator) and its inhibitor PAI-1 are statistically independent, strong prognostic factors for disease-free (DFS) and overall survival (OS). However, statistical analyses performed for varying follow-up periods suggested a time variation of prognostic strength. We therefore investigated the time-dependent prognostic power of uPA, PAI-1 and steroid hormone receptor status applying the time-varying coefficient model of Gray. uPA and PAI-1 were analysed by enzyme-linked immunosorbent assay in tumour tissue extracts from 314 breast cancer patients. Hormone receptors (oestrogen and progesterone) were determined by radioligand binding or by immunohistochemistry. Univariate and multivariate analyses (Cox proportional hazards model) of DFS and OS were performed for all patients, including 147 node-negative patients. Median follow-up of patients still alive at time of analysis (n = 232) was 58 months. Although initially of high prognostic impact, a continuous decrease over time in the prognostic power of hormone receptor status and uPA was observed. In contrast, the prognostic impact of PAI-1 increased over time and reached similar strength as the lymph node status. The time-dependent risk profile of prognostic factors may have important clinical implications in regard to follow-up and patients' individual risk situation. Evaluation of time dependency of prognostic factors may also give a more profound insight into the dynamics of breast cancer metastasis.


*British Journal of Cancer* (1997) **76**(3), 306–311
© 1997 Cancer Research Campaign

### Time-varying prognostic impact of tumour biological factors urokinase (uPA), PAI-1 and steroid hormone receptor status in primary breast cancer

**M Schmitt[1], C Thomssen[1], K Ulm[2], A Seiderer[2], N Harbeck[1], H Höfler[3], F Jänicke[1] and H Graeff[1]**

[1]Frauenklinik und Poliklinik; [2]Institut für Medizinische Statistik und Epidemiologie and [3]Institut für Allgemeine Pathologie und Pathologische Anatomie der Technischen Universität München, Ismaninger Str. 22, D-81675 Munich, Germany

**Summary** In breast cancer, several investigations have demonstrated that the tumour biological factors uPA *u*rokinase-type *p*lasminogen *a*ctivator) and its inhibitor PAI-1 are statistically independent, strong prognostic factors for disease-free (DFS) and overall survival (OS). However, statistical analyses performed for varying follow-up periods suggested a time variation of prognostic strength. We therefore investigated the time-dependent prognostic power of uPA, PAI-1 and steroid hormone receptor status applying the time-varying coefficient model of Gray. uPA and PAI-1 were analysed by enzyme-linked immunosorbent assay in tumour tissue extracts from 314 breast cancer patients. Hormone receptors (oestrogen and progesterone) were determined by radioligand binding or by immunohistochemistry. Univariate and multivariate analyses (Cox proportional hazards model) of DFS and OS were performed for all patients, including 147 node-negative patients. Median follow-up of patients still alive at time of analysis ($n$ = 232) was 58 months. Although initially of high prognostic impact, a continuous decrease over time in the prognostic power of hormone receptor status and uPA was observed. In contrast, the prognostic impact of PAI-1 increased over time and reached similar strength as the lymph node status. The time-dependent risk profile of prognostic factors may have important clinical implications in regard to follow-up and patients' individual risk situation. Evaluation of time dependency of prognostic factors may also give a more profound insight into the dynamics of breast cancer metastasis.

**Keywords:** prognosis, breast cancer, steroid hormone receptor, urokinase-type plasminogen activator, PAI-1, urokinase, protease, time variation

Breast cancer is a heterogeneous disease showing great variability of biological and clinical behaviour. The high prevalence of this disease in developed countries has stimulated vivid interest in the exploration and validation of those tumour biological parameters that may identify patients at risk. In this respect, determination of the tumour biological factors uPA (urokinase-type plasminogen activator) and its inhibitor PAI-1 is an important issue to address, as there is substantial evidence that high concentrations of these factors in primary cancer tissue are conducive to tumour cell spread and metastasis in breast cancer patients (Duffy et al, 1988; 1990; Jänicke et al, 1990; 1991; 1993; 1994; Foekens et al, 1992; 1994; Grondahl-Hansen et al, 1993; Duggan et al, 1995).

uPA and PAI-1 are strong and independent prognostic factors in both node-negative and node-positive patients; elevated antigen levels of uPA or PAI-1 are correlated with short disease-free survival and early death (Duffy et al, 1990; Jänicke et al, 1993; 1994; Foekens et al, 1992; 1994; Grondahl-Hansen et al, 1993; Duggan et al, 1995). Statistical analyses performed at different times of follow-up, however, suggest that the prognostic strength varies with time (Jänicke et al, 1990; 1991; 1993; 1994; Altman et al, 1994). Here, we analyse the time-varying impact of uPA and PAI-1 in addition to that of the traditional biological prognostic factor, steroid hormone receptor status, on breast cancer prognosis applying the time-varying coefficient model of Gray (1992). In this model, the effect of variables on relative risk (e.g. in disease-free survival) is assessed by employing an extension of the Cox proportional hazards model (Cox, 1972): the coefficient $\beta$, which in the Cox model is assumed to be constant in time, is replaced by the time-varying function $\beta(t)$.

*Received 30 September 1996*
*Accepted 9 January 1997*

*Correspondence to:* M Schmitt

### PATIENTS AND METHODS

### Patients

In a prospective observational study (1987–91), 314 patients with invasive breast cancer were enrolled (Table 1). Fresh tumour tissue was dissected by the pathologist and representative samples were snap frozen for subsequent determination of uPA and PAI-1 antigen content. Laboratory and patient data were documented in a computerized database after completion of primary therapy. Follow-up data were obtained every 3 months. Primary treatment was by modified radical mastectomy or by breast-conserving surgery, including axillary lymph node dissection. Patients with distant metastases at the time of primary surgery (M1) were not included in the study. The median number of axillary lymph nodes removed was 18 (5–45) and the median tumour diameter 2.5 cm (0.5–15 cm). The decision as to whether chemotherapeutic or hormonal adjuvant therapy should be applied was made strictly without consideration of uPA and PAI-1 antigen tissue levels and solely based on the general consensus at the time of treatment. Patients with axillary lymph node involvement received either

**Table 1** Patient's characteristics ($n = 314$)

| Variable | Frequency | (%) |
|---|---|---|
| Menopausal status | | |
| Pre/perimenopausal | 118 | 37.6 |
| Post-menopausal | 196 | 62.4 |
| Age (years) | | |
| Median | 56 | |
| Range | 27–89 | |
| Adjuvant therapy | | |
| Chemotherapy | 62 | 19.7 |
| Hormone therapy | 108 | 34.4 |
| Both | 8 | 2.6 |
| No adjuvant therapy | 136 | 43.3 |
| Tumours | | |
| Size (cm) | | |
| T1 ≤ 2 | 103 | 32.8 |
| T2 > 2 ≤ 5 | 171 | 54.5 |
| T3 > 5 | 40 | 12.7 |
| Axillary lymph node status | | |
| Negative | 147 | 46.8 |
| Positive | 167 | 53.2 |
| Histological type | | |
| Invasive ductal carcinoma | 265 | 84.4 |
| Invasive lobular carcinoma | 34 | 10.8 |
| Others | 15 | 4.8 |
| Steroid hormone receptor status | | |
| Positive | 240 | 76.4 |
| Negative | 74 | 23.6 |
| Oestrogen receptor status | | |
| Positive | 237 | 75.5 |
| Negative | 77 | 24.5 |
| Progesterone receptor status | | |
| Positive | 205 | 65.3 |
| Negative | 109 | 34.7 |
| Grading (Scarff–Bloom–Richardson) | | |
| G1/2 | 191 | 60.8 |
| G3/4 | 123 | 39.2 |
| Vessel invasion | | |
| Absent | 252 | 80.3 |
| Present | 62 | 19.7 |
| Tumour necrosis | | |
| Absent | 216 | 68.8 |
| Present | 98 | 31.2 |

adjuvant chemotherapy ($n = 66$) and/or adjuvant hormone therapy with tamoxifen (post-menopausal patients, $n = 99$); 11 node-positive patients received no form of adjuvant treatment. In the node-negative group, 125 patients did not receive any kind of adjuvant treatment; five patients were subjected to chemotherapy and 17 patients received tamoxifen (see Table 1). Chemotherapy consisted of six cycles of CMF (cyclophosphamide 600 mg $m^{-2}$ i.v. on day 1, methotrexate 40 mg $m^{-2}$ i.v. on day 1, 5-fluorouracil 600 mg $m^{-2}$ i.v. on day 1, repeated every 21 days) and hormone therapy consisted of 30 mg of tamoxifen p.o. daily. The patients were followed up by clinical visits for 5–93 months (median 52 months) at fixed intervals. Within this time of observation, 102 patients relapsed and 82 patients died. The median duration of follow-up in patients still alive at time of analysis was 58 months (range 32–93).

### Assays uPA and PAI-1 antigen were determined by a commercially available ELISA in extracts of breast cancer tissue specimens (uPA, Imubind no. 894; PAI-1; Imubind no. 821; both from American Diagnostica, Greenwich, CT, USA) and expressed as ng of antigen per mg of tissue protein; steroid hormone receptors (oestrogen and progesterone receptors) were determined in the cytosol fractions ($n = 294$) (Jänicke et al, 1990; 1991; 1993; 1994). Specimens were considered oestrogen or progesterone positive if they contained at least 20 fmol per mg of protein. In 20 tumours, immunohistochemical staining on paraffin-embedded tissue sections was performed; positive staining denoted receptor positivity.

### Statistical analysis

The prognostic impact of uPA and PAI-1 (disease-free and overall survival) was first analysed by the Cox proportional hazards model (Cox, 1972) using the SPSS software package (SPSS, Chicago, IL, USA) and by the CART *C*lassification *a*nd *R*egression *T*rees) technique (Breiman et al, 1984). For this statistical analysis, continuous as well as discrete breast cancer-related covariates were included, all of which were considered as fixed (not time dependent). Determination of the optimum cut-off for uPA and PAI-1 to discriminate low-risk and high-risk patients was performed using log-rank statistics and isotonic regression (Jänicke et al, 1990; 1991; 1993; 1994). The values with maximal log-rank test were taken for this discrimination. Group-oriented curves for disease-free and overall survival were calculated according to Kaplan and Meier (1958). The relative risks associated with the various prognostic variables after discrimination into high-risk and low-risk groups were estimated by the Cox proportional hazards model. All tests were performed at a significance level of $\alpha = 0.05$. The influence of adjuvant systemic therapy was tested by carrying out an additional Cox proportional hazard analysis including chemotherapy and hormone therapy as 'prognostic variables'. The Cox proportional hazards model is based on the assumption that the relative risk (RR) associated with a factor $x$ compared with a factor value $x = 0$ is described by $RR(x) = \exp(\beta x)$, with the coefficient $\beta$ independent of time. Prognostic factors may, however, have a changing influence on disease-free or overall survival probability with time (Gray, 1992; Lipponen et al, 1992; Yoshimoto et al, 1993). To reveal the time-varying effect of uPA, PAI-1, the steroid hormone receptor status and the axillary lymph node status on breast cancer prognosis, the extended Cox proportional hazards model of Gray was applied. In this time-varying coefficient model, the time constancy assumption on $\beta$ is relaxed and it is allowed to be a function of time, $\beta(t)$. The relative risk associated with a factor $x$ is thus modelled by $RR(t,x) = \exp(\beta(t)x)$. If $x$ is a binary variable, this expression reduces to $RR(t) = \exp(\beta(t))$. The function $\beta(t)$ is obtained by estimating $\beta$ at numerous intervals in time and then smoothing over these pointwise estimates using spline functions. These analyses were performed with the S-Plus statistical software package (Statistical Sciences, 1993).

### RESULTS

Breast cancer patients with high levels of either uPA (> 3 ng $mg^{-1}$ protein) or PAI-1 (> 14 ng $mg^{-1}$ protein) in their primary tumours (Jänicke et al, 1991) have a statistically significant increased risk

© Cancer Research Campaign 1997

**Table 2** Univariate and multivariate analyses (Cox proportional hazards model) of prognostic factors in breast cancer patients

| | Disease-free survival | | | Overall survival | | |
|---|---|---|---|---|---|---|
| | *P*-value | | Relative risk (95 % CI[a]) | *P*-value | | Relative Risk (95 % CI[a]) |
| | Univariate | Multivariate | | Univariate | Multivariate | |
| *All patients* (*n* = 314)[b] | | | | | | |
| Lymph node status (positive vs negative) | < 0.0001 | < 0.0001 | 3.0 (1.9–4.6) | < 0.0001 | < 0.0001 | 3.0 (1.8–4.9) |
| PAI-1 (ng mg$^{-1}$ protein) (> 14 vs ≤ 14) | < 0.0001 | < 0.0001 | 2.8 (1.9–4.2) | < 0.0001 | < 0.0001 | 3.5 (2.2–5.4) |
| Hormone receptor status (negative vs positive) | 0.0015 | 0.0340 | 1.6 (1.0–2.5) | < 0.0001 | 0.0016 | 2.1 (1.3–3.3) |
| *Node-negative patients* (*n* = 147)[c] | | | | | | |
| PAI-1 (ng mg$^{-1}$ protein) (> 14 vs ≤ 14) | < 0.0001 | < 0.0001 | 4.9 (2.3–10.6) | < 0.0001 | < 0.0001 | 9.5 (3.8–23.8) |

[a]CI = confidence interval.[b]The following variables were also of statistical significance judged by univariate analysis and thus included in the Cox model: vessel invasion, grading, tumour necrosis, uPA and tumour size. None of these variables was of statistical significance in multivariate analysis. [c]uPA (disease-free survival) and vessel invasion (overall survival) were also statistically significant in univariate analysis and thus included in the Cox model. In multivariate analysis uPA and vessel invasion failed to reach statistical significance. Steroid hormone receptor status, grade, menopausal status, tumour size, presence of tumour necrosis and histological type were not statistically significant in univariate analysis.

**Figure 1** Disease-free survival of 147 node-negative breast cancer patients and time variation of the relative risks of disease recurrence in relation to steroid hormone receptor status (**A**), uPA antigen level (**B**) and PAI-1 antigen level (**C**). PAI-1, uPA and the steroid hormone receptor status were used as dichotomized covariates: steroid hormone receptor-negative vs receptor-positive, uPA > 3 ng vs ≤ 3 ng mg$^{-1}$ protein, PAI-1 > 14 ng vs ≤ 14 ng mg$^{-1}$ protein. Disease-free survival, depicted on the left, was estimated by the Kaplan–Meier method. Time variation of the relative risk of disease recurrence, depicted on the right, was calculated by the time-varying coefficient model of Gray (1992) and function plots (observation time vs relative risk) were constructed for each risk factor within a time frame of 6–42 months (shaded area). (- - -), 95% confidence interval. NS, not significant

© Cancer Research Campaign 1997

**Figure 2** Time variation of the relative risks of disease recurrence in relation to uPA, PAI-1, steroid hormone receptor status and axillary lymph node status in 314 breast cancer patients. The time-varying coefficient model of Gray (1992) was applied. PAI-1, uPA, axillary lymph node status (positive vs negative) and steroid hormone receptor status were included in the model as dichotomized covariates and a function plot (observation time vs relative risk) displayed. The vertical bars at the top of the plot represent a frequency plot of recurrences of disease recurrence and death as judged by univariate analyses (Table 2). In order to weight uPA and PAI-1 with traditional prognostic factors (axillary lymph node status, steroid hormone receptor status, grade, vessel invasion, tumour size, and presence of tumour necrosis), we made use of the Cox proportional hazards model for both disease-free and overall survival. Only axillary lymph node status, PAI-1 and steroid hormone receptor status turned out to be independent and strong prognostic factors. In node-negative patients, PAI-1 is the only independent and strong prognostic factor (Table 2).

In order to find out why uPA and steroid hormone receptor status, but not PAI-1, had lost their statistically independent prognostic power at the time of analysis, we analysed the prognostic influence of uPA, PAI-1, and steroid hormone receptor status in our group of node-negative patients by applying the time-varying coefficient model of Gray (1992). The evaluation of this model was confined to the first 42 months of observation after primary treatment because only during this time interval were sufficient numbers of patients and recurrences available for this type of statistical analysis.

The relative risk of disease recurrence evolves as a function of time for all three parameters evaluated (Figure 1). We noticed that, during the period of observation, the prognostic power of the steroid hormone receptor status decreased steadily, even falling below a relative risk = 1 after 2 years of follow-up (Figure 1A). This result illustrates that, if patients with steroid hormone receptor-negative breast cancer survive the first 2 years after primary treatment without experiencing a recurrence, they have an up to fivefold higher probability of being cured than patients with steroid hormone receptor-positive tumours. This finding is illustrated in the course of the Kaplan–Meier plot of steroid hormone receptor status. The curves for disease-free survival of the low-risk and high-risk group begin to converge after a follow-up of 3 years. For uPA, we found a similar result: high uPA-dependent relative risks indicate early recurrences, whereas around 32 months after primary treatment the relative risk of recurrence decreases to values close to 1 (Figure 1B). Evidently, after this time, uPA has lost its independent prognostic power. The time-varying impact of PAI-1 is opposite to that of uPA and steroid hormone receptor status (Figure 1C). The prognostic impact of PAI-1 increases with observation time, reaching a peak level at about 33 months. Evidently, for uPA and steroid hormone receptor status, high relative risks indicate early disease recurrence whereas a high PAI-1 risk profile is associated with later relapse.

A similar trend as displayed in Figure 1 for node-negative patients is demonstrated in Figure 2 for the entire group of breast cancer patients including those with node-negative and node-positive disease. Initially, at 6 months, the axillary lymph node status turns out to be of highest prognostic impact. This effect levels off slightly over time. Similar to node-negative breast cancer, both steroid hormone receptor status and uPA indicate early disease recurrence, in contrast to the time-varying impact of PAI-1, which increases with the observation time, reaching similar strength in predicting disease-free survival as the axillary lymph node status.

### DISCUSSION

Time dependency of the impact of axillary lymph node and steroid hormone receptor status on prognosis in breast cancer patients was first shown by Gray in 1992. We have applied his modified Cox model to study the time-varying impact on prognosis of the new tumour biological factors uPA (urokinase-type plasminogen activator) and PAI-1 (inhibitor type 1 to uPA) and also included the traditional prognostic factors, axillary lymph node status and steroid hormone receptor status, in this analysis. The power of uPA to predict recurrences was strong in the first 3 years, especially in node-negative patients, but declined substantially thereafter. This time-related pattern of uPA was similar to that already described for steroid hormone receptor status.

In contrast, the prognostic impact of PAI-1 increases over time and becomes even stronger in predicting disease-free survival than axillary lymph node status. On the other hand, the relative risk for recurrence, as indicated by axillary lymph node status, after an initial decline does not vary essentially throughout the observation period. In node-negative breast cancer patients, PAI-1 even prevails as the only independent prognostic factor in our analysis. Confounding effects of adjuvant therapy might have influenced these results. Thus, adjuvant therapy was included as a prognostic variable in the statistical analysis. However, the application of adjuvant therapy was not an independent risk factor. An interaction between prognostic factors and adjuvant therapy affecting the outcome of the statistical analysis could therefore be excluded.

Invasion and metastasis of solid tumours require the degradation of the extracellular matrix and of the basement membrane at the site of the primary tumour and at distant loci. Hence, proteolysis and remodelling of the matrix at the metastatic site are essential. Proteases such as uPA, matrix metalloproteases, cathepsins, plasmin and thrombin and their inhibitors are involved in these processes (Schmitt et al, 1992; Brünner et al, 1994). Efficient tumour cell invasiveness, focal proteolysis and metastasis with secondary tumour growth are based on a critical balance between proteases, their cell-surface receptors and inhibitors (Liu et al, 1995). Tumour biological studies have attributed a key role to uPA, its cell-surface-bound receptor (uPA-R) and PAI-1 in these events. The overexpression of uPA-R in breast cancer cells results in increased tumour invasion and metastasis in an experimental model (Xing and Rabbani, 1996). uPA-R is a major binding protein to the vitronectin-rich extracellular matrix (Kanse et al, 1996). It may in addition regulate the $\beta_1$-integrin function, thus

© Cancer Research Campaign 1997

influencing cell adhesion and direct migration of adherent cells (Felsenfeld et al, 1996; Wei et al, 1996). Indeed, in addition to breast cancer, a strong impact on prognosis of uPA content in tumour tissue has been observed in many other malignancies, e.g. cancer of the ovary (Kuhn et al, 1994), stomach (Nekarda et al, 1994; Heiss et al, 1995), colon (Ganesh et al, 1994), lung (Pedersen et al, 1994), kidney (Hofmann et al, 1996), bladder (Hasui et al, 1989) and cervix uteri (Kobayashi et al, 1994). It is rather difficult to understand that the tumour tissue content of the uPA inhibitor PAI-1 on an even larger scale indicates a poor prognosis for the cancer patient. Suggestions to explain these findings are that PAI-1 is a prerequisite for the matrix formation at the metastatic site by protecting the tumour against tumour-associated proteases (Sier et al, 1994). Recent observations indicate an even broader role for PAI-1 in tumour biology. It may be involved in the modulation of the uPA-R binding to vitronectin (Kanse et al, 1996) and is thought to inhibit cell attachment to the extracellular matrix (Stefansson and Lawrence, 1996), thus enabling tumour cells to migrate in a stepwise fashion, alternately being attached to the extracellular matrix or detached from it. These findings underline the strong effect of PAI-1 on the malignant phenotype of the tumour cell and are in line with the observation that coexpression of uPA, its receptor and PAI-1 is necessary for focalized and optimal invasiveness (Estreicher et al, 1990; Liu et al, 1995), as well as for angiogenic activity (Barbareschi et al, 1995).

Shedding and dissemination of tumour cells is a very common phenomenon in solid tumours, especially in breast cancer. The presence of tumour cells distant from the primary tumour, however, is not a definite sign for later occurrence of distant metastases. So-called dormant micrometastases may be present for years and may even disappear spontaneously or develop angiogenic activity and switch to the expanding, invasive phenotype by different, unknown stimuli (Holmgren et al, 1995). These stimuli may also involve, among other things, the plasminogen-activating system mediated by the tumour cell surface-located receptor for uPA (uPA-R). Indeed, Heiss et al (1995) in a recent study demonstrated that the presence of uPA-R on disseminated tumour cells detected in bone marrow aspirates of gastric cancer patients is a strong indicator for the development of later clinical metastases. Thus, the occurrence of clinically detectable metastases seems to depend on the biological properties of the disseminated tumour cell. One might speculate that tumour cells exhibiting a high capacity to synthesize uPA are primarily of the invasive phenotype or will switch early during the course of disease to invasiveness, whereas those producing high levels of PAI-1 will become invasive at a later time. Studying the time variation of the risk associated with these and other factors may give important insights into their role in tumour cell dissemination and metastasis.

Our findings may still be well short of changing clinical practice at the moment. However, both the absence of steroid hormone receptors and high uPA tumour levels in breast cancer patients can be said to be indicators of early disease recurrence. In patients with steroid hormone receptor-negative tumours or tumours with high uPA content, who remain disease free during the first 2 years of follow-up, late recurrence tends to be rare. On the other hand, patients with high PAI-1 tumour levels have the highest relative risk of recurrence during the second and third years. In conclusion, the knowledge of the time-dependent risk profile of prognostic factors in breast cancer might have important clinical implications regarding follow-up and the assessment of the patient's individual risk situation.

### ACKNOWLEDGEMENTS

This study was supported by the Deutsche Forschungsgemeinschaft (Klinische Forschergruppe GR280/4-1, GR280/4-2, GR280/4-5), the Wilhelm Sander-Stiftung and by a grant from the European Union (BIOMED-1, BMH1-CT93-1346). The authors thank L Pache MD, A Schäfer MD and A Prechtl MD for help with the statistical evaluation and follow-up of patients and E. Sedlaczek, B Jaud-Münch, and H Seibold for expert technical assistance. The generous supply of ELISA kits for uPA and PAI-1 by R Hart PhD, American Diagnostica Inc., Greenwich, CT, USA, is gratefully acknowledged.

### REFERENCES

Altman DG, Lausen B, Sauerbrei W and Schumacher M (1994) Dangers of using 'optimal' cutpoints in the evaluation of prognostic factors. *J Natl Cancer Inst* **86**: 829–835

Barbareschi M, Gasparini G, Morelli L, Forti S and Della-Palma P (1995) Novel methods for the determination of the angiogenic activity of human tumors. *Breast Cancer Res Treat* **36**: 181–192

Breiman L, Friedman JH and Olsen RA (1984) *Classification and Regression Trees.* Wadsworth: Belmont

Brünner N, Pyke C, Hansen CH, Romer J, Grøndahl-Hansen J and Danø K (1994) Urokinase plasminogen activator (uPA) and its type 1 inhibitor (PAI-1): regulators of proteolysis during cancer invasion and prognostic parameters in breast cancer. *Cancer Treat Res* **71**: 299–309

Cox DR (1972) Regression models and life-tables. *J R Stat Soc (B)* **34**: 187–200

Duffy M, O'Grady P, Devaney D, O'Siorain L, Fennelly JJ and Lijnen HJ (1988). Urokinase-plasminogen activator, a marker for aggressive breast carcinomas. *Cancer* **62**: 531–533

Duffy MJ, Reilley D, O'Sullivan C, O'Higgins N, Fennelly JN and Andreasen P (1990) Urokinase-plasminogen activator, a new and independent prognostic marker in breast cancer. *Cancer Res* **50**: 6827–6829

Duggan C, Maguire T, McDermott E, O'Higgins N, Fennelly JJ and Duffy MJ (1995) Urokinase plasminogen activator and urokinase plasminogen activator receptor in breast cancer. *Int J Cancer* **61**: 597–600

Estreicher A, Muhlhauser J, Carpentier JL, Orci L and Vassalli JD (1990) The receptor for urokinase type plasminogen activator polarizes expression of the protease to the leading edge of migrating monocytes and promotes degradation of enzyme inhibitor complexes. *J Cell Biol* **111**: 783–792

Felsenfeld DP, Choquet D and Sheetz MP (1996) Ligand binding regulates the directed movement of $\beta_1$ integrins on fibroblasts. *Nature* **383**: 438–440

Foekens JA, Schmitt M, van Putten WLJ, Peters HA, Jänicke F and Klijn JMG (1994) Plasminogen activator inhibitor-1 and prognosis in primary breast cancer. *J Clin Oncol* **12**: 1648–1658

Foekens JA, Schmitt M, van Putten WLJ, Peters HA, Bontenbal M, Jänicke F and Klijn JGM (1992) Prognostic value of urokinase-type plasminogen activator in 671 primary breast cancer patients. *Cancer Res* **52**: 6101–6105

Ganesh S, Sier CF, Griffioen G, Vloedgraven HJ, de Boer A, Welvaart K, van de Velde CJ, van Krieken JH, Verheijen JH, Lamers CB and Verspaget HW (1994) Prognostic relevance of plasminogen activators and their inhibitors in colorectal cancer. *Cancer Res* **54**: 4065–4071

Gray RJ (1992). Flexible methods for analyzing survival data using splines, with applications to breast cancer prognosis. *J Am Stat Assoc* **87**: 942–951

Grøndahl-Hansen J, Christensen IJ, Rosenquist C, Rosenquist C, Brünner N, Mouridsen HT, Danø K and Blichert-Toft M (1993) High levels of urokinase-type plasminogen activator (uPA) and its inhibitor PAI-1 in cytosolic extracts of breast carcinomas are associated with poor prognosis. *Cancer Res* **53**: 2513–2521

Hasui Y, Suzumiya J, Marutsuka K, Sumiyoshi A, Hashida S and Ishikawa E (1989) Comparative study of plasminogen activators in cancers and normal mucosae of human urinary bladder. *Cancer Res* **49**: 1067–1070

Heiss MM, Allgayer H, Gruetzner KU, Funke I, Babic R, Jauch KW and Schildberg FW (1995) Individual development and uPA-receptor expression of disseminated tumor cells in bone marrow: A reference to early systemic disease in solid cancer. *Nature Med* **1**: 1035–1039

Heiss MM, Babic R, Allgayer H, Gruetzner KU, Jauch KW, Loehrs U and Schildberg FW (1995) Tumor associated proteolysis and prognosis: new

© Cancer Research Campaign 1997

functional risk factors in gastric cancer defined by the urokinase type plasminogen activator system. *J Clin Oncol* **13**: 2084–2093

Hofmann R, Lehmer A, Hartung R, Robrecht C, Buresch M and Grothe F (1996) Prognostic value of urokinase plasminogen activator and plasminogen activator inhibitor-1 in renal cell cancer. *J Urol* **155**: 858–862

Holmgren L, O'Reilly MS and Folkman J (1995) Dormancy of micrometastases: Balanced proliferation and apoptosis in the presence of angiogenesis suppression. *Nature Med* **2**: 149–153

Jänicke F, Pache L, Schmitt M, Ulm K, Thomssen Ch, Prechtl A and Graeff H (1994) Both the cytosols and detergent extracts of breast cancer tissues are suited to evaluate the prognostic impact of the urokinase-type plasminogen activator and its inhibitor plasminogen activator inhibitor type 1. *Cancer Res* **54**: 2527–2530

Jänicke F, Schmitt M and Graeff H (1991) Clinical relevance of the urokinase-type and the tissue-type plasminogen activators and of their inhibitor PAI-1 in breast cancer. *Semin Thromb Hemostas* **17**: 303–312

Jänicke F, Schmitt M, Hafter R, Hollrieder A, Babic R, Ulm K, Gössner W and Graeff H (1990) Urokinase-type plasminogen activator (u-PA) antigen is a predictor of early relapse in breast cancer. *Fibrinolysis* **4**: 69–78

Jänicke F, Schmitt M, Pache L, Ulm K, Harbeck N, Höfler H and Graeff H (1993) Urokinase (uPA) and its inhibitor PAI-1 are strong, independent prognostic factors in node-negative breast cancer. *Breast Cancer Res Treat* **24**: 195–208

Kanse S, Kost C, Wilhelm O, Andreasen PA and Preissner KT (1996) The urokinase receptor is a major vitronectin-binding protein on endothelial cells. *Exp Cell Res* **224**: 344–353

Kaplan EL and Meier P (1958) Nonparametric estimation from incomplete observations. *J Am Stat Assoc* **53**: 457–481

Kobayashi H, Fujishiro S and Terao T (1994) Impact of urokinase type plasminogen activator and its inhibitor type 1 on prognosis in cervical cancer of the uterus. *Cancer Res* **54**: 6539–6548

Kuhn W, Pache L, Schmalfeldt B, Dettmar P, Schmitt M, Jänicke F and Graeff H (1994) Urokinase (uPA) and PAI-1 predict survival in advanced ovarian cancer patients (FIGO III) after radical surgery and platinum-based chemotherapy. *Gynecol Oncol* **55**: 401–409

Lipponen P, Aaltomaa S, Eskelinen M, Kosma VM, Marin S and Syrjanen K (1992) The changing importance of prognostic factors in breast cancer during long-term follow-up. *Int J Cancer* **51**: 698–702

Liu G, Shuman MA and Cohen RL (1995) Co-expression of urokinase, urokinase receptor and PAI-1 is necessary for optimum invasiveness of cultured lung cancer cells. *Int J Cancer* **60**: 501–506

Nekarda H, Schmitt M, Ulm K, Wenninger A, Vogelsang H, Becker K, Roder JD, Fink U and Siewert JR (1994) Prognostic impact of urokinase-type plasminogen activator and its inhibitor PAI-1 in completely resected gastric cancer. *Cancer Res* **54**: 2900–2907

Pedersen H, Brünner N, Francis D, Osterlind K, Rønne E, Hansen HH, Danø K and Grondahl-Hansen J (1994) Prognostic impact of urokinase, urokinase receptor and type 1 plasminogen activator inhibitor in squamous and large cell lung cancer tissue. *Cancer Res* **54**: 4671–4675

Schmitt M, Jänicke F and Graeff H (1992) Tumor-associated proteases. *Fibrinolysis* **4** (suppl.): 3–26

Sier CM, Vloedgraven HJM, Ganesh S, Griffioen G, Quax PH, Verheijen JH, Dooijewaard G, Welvaart K, van de Velde CJ, Lamers CB and Verspaget HW (1994) Inactive urokinase and increased levels of its inhibitor type 1 in colorectal cancer liver metastasis. *Gastroenterology* **107**: 1449–1456

Statistical Sciences (1993) S-PLUS *Guide to Statistical and Mathematical Analysis*, Version 3.2. StatSci: Seattle, WA

Stefansson S and Lawrence DA (1996) The serpin PAI-1 inhibits cell migration by blocking integrin $\alpha_v\beta_3$ binding to vitronectin. *Nature* **383**: 441–443

Wei Y, Lukashev M, Simon DI, Bodary SC, Rosenberg S, Doyle MV and Chapman HA (1996) Regulation of integrin function by the urokinase receptor. *Science* **273**: 1551–15

Xing RH and Rabbani SA (1996) Overexpression of urokinase receptor in breast cancer cells results in increased tumor invasion, growth and metastasis. *Int J Cancer* **67**: 423–429

Yoshimoto M, Sakamoto G and Ohashi Y (1993) Time dependency of the influence of prognostic factors on relapse in breast cancer. *Cancer* **10**: 2993–3001

© Cancer Research Campaign 1997